# Plasma Modification of Poly Lactic Acid Solutions to Generate High Quality Electrospun PLA Nanofibers

**DOI:** 10.1038/s41598-018-20714-5

**Published:** 2018-02-02

**Authors:** Fatemeh Rezaei, Anton Nikiforov, Rino Morent, Nathalie De Geyter

**Affiliations:** Research Unit Plasma Technology (RUPT), Department of Applied Physics, Faculty of Engineering and Architecture, Ghent University, St-Pietersnieuwstraat 41 B4, 9000 Ghent, Belgium

## Abstract

Physical properties of pre-electrospinning polymer solutions play a key role in electrospinning as they strongly determine the morphology of the obtained electrospun nanofibers. In this work, an atmospheric-pressure argon plasma directly submerged in the liquid-phase was used to modify the physical properties of poly lactic acid (PLA) spinning solutions in an effort to improve their electrospinnability. The electrical characteristics of the plasma were investigated by two methods; V-I waveforms and Q-V Lissajous plots while the optical emission characteristics of the plasma were also determined using optical emission spectroscopy (OES). To perform a complete physical characterization of the plasma-modified polymer solutions, measurements of viscosity, surface tension, and electrical conductivity were performed for various PLA concentrations, plasma exposure times, gas flow rates, and applied voltages. Moreover, a fast intensified charge-couple device (ICCD) camera was used to image the bubble dynamics during the plasma treatments. In addition, morphological changes of PLA nanofibers generated from plasma-treated PLA solutions were observed by scanning electron microscopy (SEM). The performed plasma treatments were found to induce significant changes to the main physical properties of the PLA solutions, leading to an enhancement of electrospinnability and an improvement of PLA nanofiber formation.

## Introduction

Atmospheric-pressure non-equilibrium plasmas have recently gained increasing attention for different applications, such as decontamination and sterilization of surfaces or living tissues^1^, surface activation^2–4^, and liquid treatment^5^. One of the emerging novel applications of these plasmas in the field of plasma-liquid interactions is modification of pre-electrospinning polymer solutions^6–8^.

Electrospinning is a fiber fabrication technique which relies on the application of electrostatic forces between a nozzle-tipped syringe containing the polymer solution and a collector for the deposition of nanofibers. As such, the polymer solution will be electrically charged and a Taylor cone will be formed at the nozzle tip. Overcoming the surface tension of the polymer solution, the electrostatic forces lead to the formation of a polymer jet from the nozzle tip towards the collector. While the polymer jet travels towards the collector, the solvents evaporate leading to nanofiber deposition on the collector. This technique has gained more interest in recent years due to the large application potential of nanofibers in various fields including aerospace, energy generation and storage, fabrication of sensitive optical sensors, filtration, IT, and biomedical applications such as tissue engineering and drug delivery^9–11^. Usually, the production of uniform and bead-free fibers with desirable properties for a specific application is not easy as multiple electrospinning parameters are known to determine the morphology of the resultant nanofibers. These parameters can be divided into three groups: electrospinning working parameters (such as applied voltage, feed rate, and working distance), properties of the electrospinning polymer solution, and ambient conditions^12^. Multiple studies have examined the effect of electrospinning working parameters and ambient conditions on numerous polymer solutions^13–15^, whereas the effect of the polymer solution properties (besides polymer concentration) on the electrospinning process have not been widely investigated. Nevertheless, the physical properties of the polymer solution such as concentration, viscosity, surface tension, and conductivity are known to be the main factors influencing the electrospinnability of the solution as well as the final fiber morphology and diameter^16^. As such, it can be said that besides selecting the right working parameters, preparation of a suitable polymer solution is also very crucial in the electrospinning process. In the past, there have been efforts to increase the electrospinnability of polymer solutions by using physical cross-linkers or by the addition of salts^17–20^. However, this approach often involves additional costs, safety concerns as well as environmental issues. Within this context, it is thus important to search for very effective, environmentally friendly and non-toxic methods which are able to improve the electrospinnability of polymer solutions.

Very recently non-equilibrium plasma technology has been taken into consideration by some research groups to modify polymer solutions before electrospinning^6–8^. Such environmentally benign plasma treatments were found to offer various advantages of which the most important one is the production of thinner and bead-free nanofibers at significantly lower polymer concentrations. For example, Shi *et al*.^6,21^ used a capacitively-coupled helium dielectric barrier discharge to treat polyethylene oxide (PEO) and Ag/polyacrylonitrile (Ag/PAN) solutions and observed that the polymer solution viscosity, conductivity, and surface tension increased after plasma treatment and consequently finer and smoother nanofibers were formed with fewer microbeads and increased crystallinity. These authors also studied the effects of plasma exposure time and polymer concentration on the final electrospinnability of the solutions. In another study by Colombo *et al*.^7^, a single electrode argon plasma jet was found to successfully promote the electrospinnability of poly-L-lactic acid (PLLA) solutions in dichloromethane. The effects of peak voltage, pulse repetition frequency, solution volume, and plasma treatment time on the final morphology of electrospun fibers were investigated in their work. In these preliminary studies, the discharges were always generated above the surface of the polymer solutions. However, the work presented in this paper will be novel as it intends to employ an atmospheric-pressure plasma jet directly submerged into the polymer solution. As such, a more pronounced plasma treatment effect on the exposed pre-electrospinning polymer solutions is anticipated. Moreover, the present work systematically investigate the effects of this directly submerged plasma treatment on the physical properties of the pre-electrospinning polymer solutions and also on the final morphology of the produced nanofibers considering various operational parameters including polymer concentrations, applied voltage, gas flow rate, and plasma treatment time. Poly lactic acid (PLA) will be considered as the target polymer in this study as it is a semi-crystalline polymer broadly used in various biomedical applications and clinical uses due to its biodegradability, biocompatibility, and good mechanical properties^22^. As such, the resultant PLA nanofibers can be applied for example as tissue engineering scaffolds.

Understanding the physical interactions between the submerged non-equilibrium plasma jet and the pre-electrospinning PLA solution is very important for the further optimization of the plasma-solution system. Therefore, the objective of this study is to evaluate the effect of plasma treatment and bubble behavior on the physical properties of the PLA solutions and to study the plasma treatment effects on the morphology of the obtained PLA nanofibrous mats. To do so, plasma treatments will be carried out making use of various exposure times, gas flow rates, and applied voltages. The argon plasma will be examined in detail making use of optical emission spectroscopy (OES) and electrical measurements while the plasma bubble dynamics will be investigated using an ICCD camera system. Additionally, the viscosity, surface tension, and electrical conductivity of PLA solutions will be measured before and after plasma treatments with varying operational parameters. In a final step, untreated and plasma-modified PLA solutions will be electrospun after which the resultant nanofiber morphology will be studied using scanning electron microscopy (SEM).

## Results

### Electrical Characterization of the Plasma Jet

**Figure 1:**
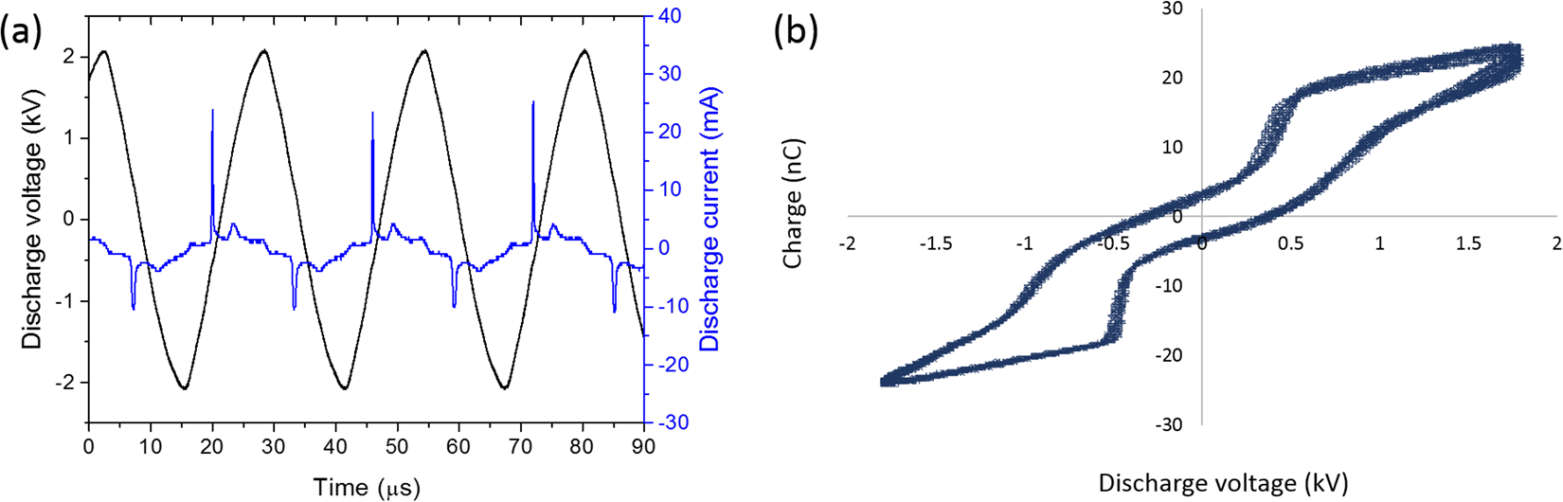
(**a**) Voltage-current graph, and (**b**) Lissajous curve showing the charge-voltage variation in the argon plasma jet (gas flow rate: 0.3 ${\rm{l}}\,{{\rm{\min }}}^{-1}$, applied voltage: 4 ${{\rm{kV}}}_{{\rm{P}}-{\rm{P}}}$).

The discharge electrical characteristics were studied using two methods; V-I waveforms and Q-V Lissajous plots. A typical voltage-current waveform plot for an applied voltage of 2 kV is shown in Fig. 1a. To obtain the mean discharge power using the first method (V-I plots), the following equation was used:

1 $${\rm{P}}=\frac{1}{{\rm{T}}}{\int }^{}{\rm{I}}({\rm{t}}){{\rm{V}}}_{{\rm{d}}}({\rm{t}}){\rm{dt}}$$

where T is the period of the discharge voltage and ${{\rm{V}}}_{{\rm{d}}}$ is the discharge voltage.

The second method for estimation of the mean discharge power includes the use of a Lissajous plot. By measuring the voltage over a capacitor (${{\rm{V}}}_{{\rm{c}}}$(t)) and the discharge voltage (${{\rm{V}}}_{{\rm{d}}}$(t)) and using multiple periods of the discharge voltage, a Q-V Lissajous characteristic curve could be plotted of which an example is shown in Fig. 1b for an applied voltage of 2 kV. This method offers an advantage as it can avoid the phase error occurring between the voltage and current measurements. After obtaining the charge on the capacitor, the mean discharge power can be determined using the following equations:

2 $$\begin{array}{c}{\rm{Q}}({\rm{t}})={{\rm{CV}}}_{{\rm{c}}}({\rm{t}}),\,{{\rm{V}}}_{{\rm{c}}}({\rm{t}})=\frac{1}{{\rm{C}}}{\int }^{}{\rm{i}}({\rm{t}})\mathrm{dt},\\ {\rm{i}}({\rm{t}})={\rm{C}}\frac{{{\rm{dV}}}_{{\rm{c}}}}{{\rm{dt}}}\mathop{\to }\limits^{\mathrm{substituting}\,\mathrm{in}\,\mathrm{Equation}\,2}{\rm{P}}=\frac{1}{{\rm{T}}}{\int }^{}{{\rm{V}}}_{{\rm{d}}}({\rm{t}}){\rm{dQ}}\end{array}$$

**Figure 2:**
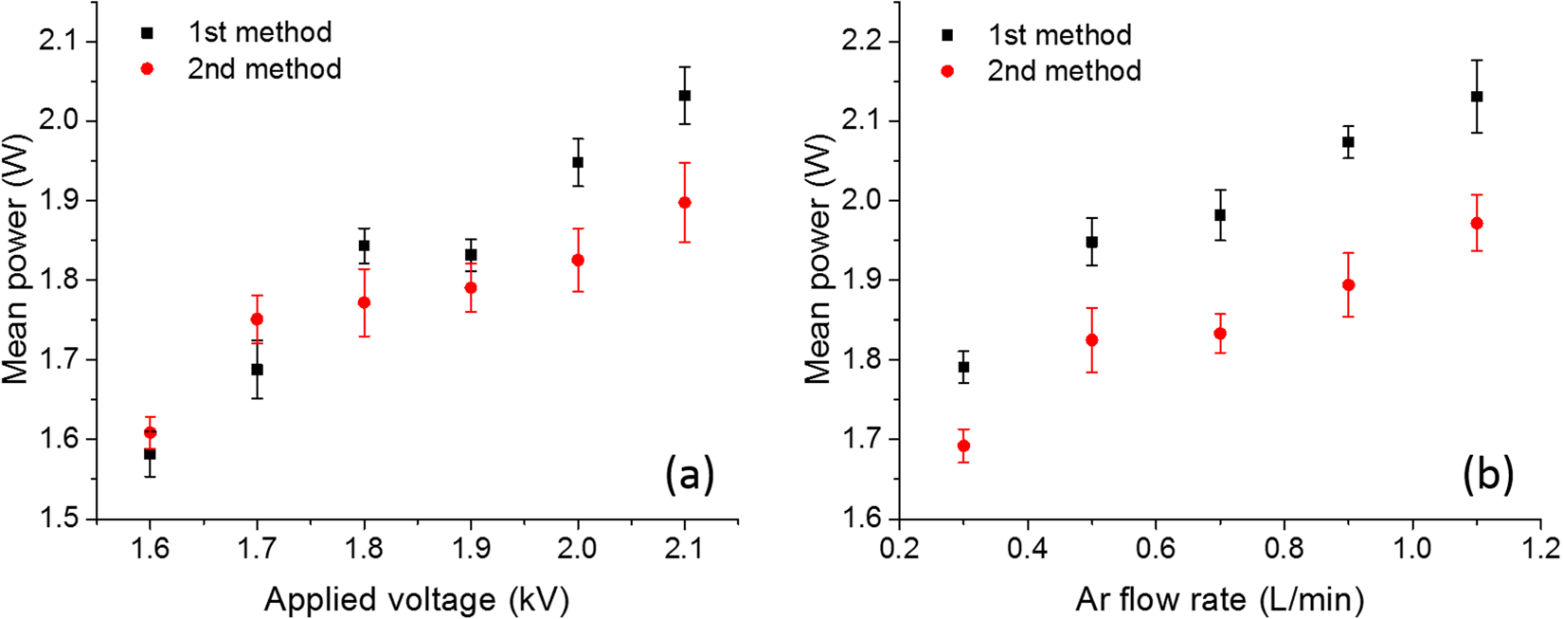
Mean consumed power obtained by two methods as a function of (**a**) applied voltages (Ar flow rate: 0.5 ${\rm{l}}\,{{\rm{\min }}}^{-1}$), and (**b**) Ar flow rates (applied voltage: 2 kV).

The obtained mean power values by the two methods as a function of discharge voltage (at fixed argon flow rate of 0.5 ${\rm{l}}\,{{\rm{\min }}}^{-1}$) and argon flow rate (at fixed applied voltage of 2 kV) applied in this study are shown in Fig. 2 and are the average of three individual measurements per condition.

### Optical Characterization of the Plasma Jet

**Figure 3:**
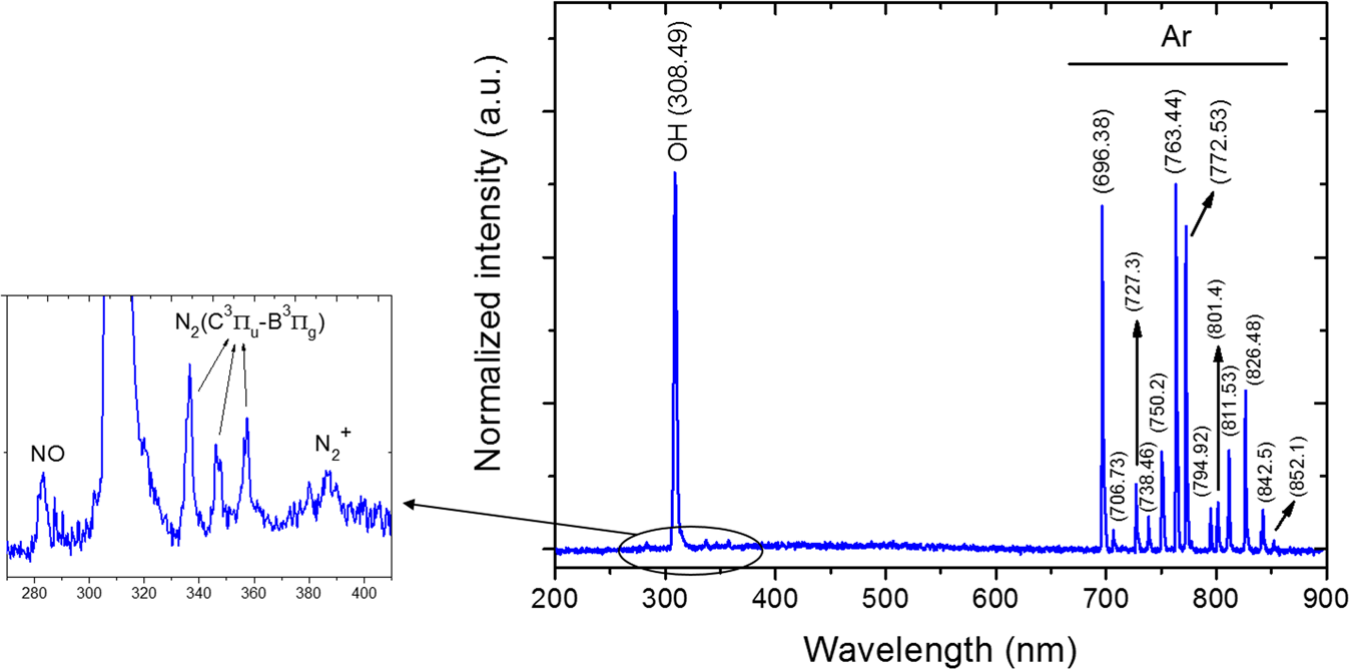
Optical emission spectrum of the plasma jet (argon flow rate: 0.5 lmin^−1^, amplitude of the applied voltage: 2 kV).

To determine the radiative plasma species present in the plasma jet, OES measurements were performed making use of an optical fiber placed at 4.5 mm from the end of the capillary tube to study the optical emission spectrum from the plasma afterglow. In Fig. 3 an optical emission spectrum of the plasma jet afterglow operating in ambient air, over the spectral range of 200–900 nm, is shown. This figure clearly shows the presence of multiple transition lines. A detailed allocation of each transition line to a specific species can be found in Table 1.

**Table 1:** Main transition lines observed in the optical emission spectrum of the argon plasma jet.

Species	Transition line	Wavelength (nm)	Ref.
Ar	$1{{\rm{s}}}_{5}\leftarrow 2{{\rm{p}}}_{2}$	696.38	^28,35^
	$1{{\rm{s}}}_{5}\leftarrow 2{{\rm{p}}}_{3}$	706.73	^28,35^
	$1{{\rm{s}}}_{4}\leftarrow 2{{\rm{p}}}_{2}$	727.30	^28,35^
	$1{{\rm{s}}}_{4}\leftarrow 2{{\rm{p}}}_{3}$	738.46	^28,35^
	$1{{\rm{s}}}_{2}\leftarrow 2{{\rm{p}}}_{1}$	750.20	^28,35^
	$1{{\rm{s}}}_{5}\leftarrow 2{{\rm{p}}}_{6}$	763.44	^28,35^
	$1{{\rm{s}}}_{3}\leftarrow 2{{\rm{p}}}_{2}$	772.53	^28,35^
	$1{{\rm{s}}}_{3}\leftarrow 2{{\rm{p}}}_{4}$	794.92	^28,35^
	$1{{\rm{s}}}_{5}\leftarrow 2{{\rm{p}}}_{8}$	801.40	^28,35^
	$1{{\rm{s}}}_{5}\leftarrow 2{{\rm{p}}}_{9}$	811.53	^28,35^
	$1{{\rm{s}}}_{2}\leftarrow 2{{\rm{p}}}_{2}$	826.48	^28,35^
	$1{{\rm{s}}}_{4}\leftarrow 2{{\rm{p}}}_{8}$	842.50	^28,35^
	$1{{\rm{s}}}_{2}\leftarrow 2{{\rm{p}}}_{4}$	852.10	^28,35^
O	$3{{\rm{s}}}^{5}{\rm{S}}\leftarrow 3{{\rm{p}}}^{5}{\rm{P}}$	777.53	^53^
OH	${{\rm{X}}}^{2}{\rm{\Pi }}\leftarrow {{\rm{A}}}^{2}{{\rm{\Sigma }}}^{+}$	308.49	^28,35^
${{\rm{N}}}_{2}$	${{\rm{B}}}^{3}{{\rm{\Pi }}}_{{\rm{g}}}\leftarrow {{\rm{C}}}^{3}{{\rm{\Pi }}}_{{\rm{u}}}$	337.26	^32^
	${{\rm{B}}}^{3}{{\rm{\Pi }}}_{{\rm{g}}}\leftarrow {{\rm{C}}}^{3}{{\rm{\Pi }}}_{{\rm{u}}}$	346.06	^32^
	${{\rm{B}}}^{3}{{\rm{\Pi }}}_{{\rm{g}}}\leftarrow {{\rm{C}}}^{3}{{\rm{\Pi }}}_{{\rm{u}}}$	357.41	^32^
	${{\rm{B}}}^{3}{{\rm{\Pi }}}_{{\rm{g}}}\leftarrow {{\rm{C}}}^{3}{{\rm{\Pi }}}_{{\rm{u}}}$	380.00	^32^
${{\rm{N}}}_{2}^{+}$	${{\rm{X}}}^{2}{{\rm{\Sigma }}}_{{\rm{g}}}^{+}\leftarrow {{\rm{B}}}^{2}{{\rm{\Sigma }}}_{{\rm{u}}}^{+}$	388.00	^54^
NO	${{\rm{X}}}^{2}{\rm{\Pi }}\leftarrow {{\rm{A}}}^{2}{{\rm{\Sigma }}}^{+}$	282.87	^55^

**Figure 4:**
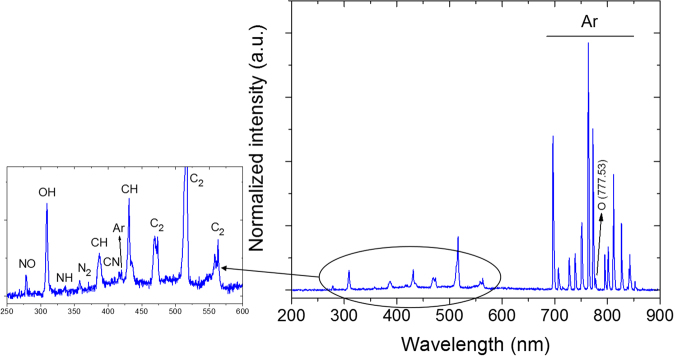
Optical emission spectrum of the plasma jet submerged in the PLA solution (argon flow rate: 0.5 lmin^−1^, amplitude of the applied voltage: 2 kV).

During the plasma treatment, the optical emission spectrum of the plasma jet submerged in the PLA solution was also extracted and is shown in Fig. 4.

### Viscosity and Conductivity of the Plasma-Treated PLA Solutions

From literature, it is well known that polymer solution viscosity is one of the key parameters in determining the fiber diameter and morphology; in addition, also the electrical conductivity of the polymer solution can strongly affect the electrospinning performance^13,23^. Therefore, these two key physical properties of PLA solutions will be analyzed in this section to assess the plasma treatment effect on these parameters making use of multiple experimental conditions.

**Figure 5:**
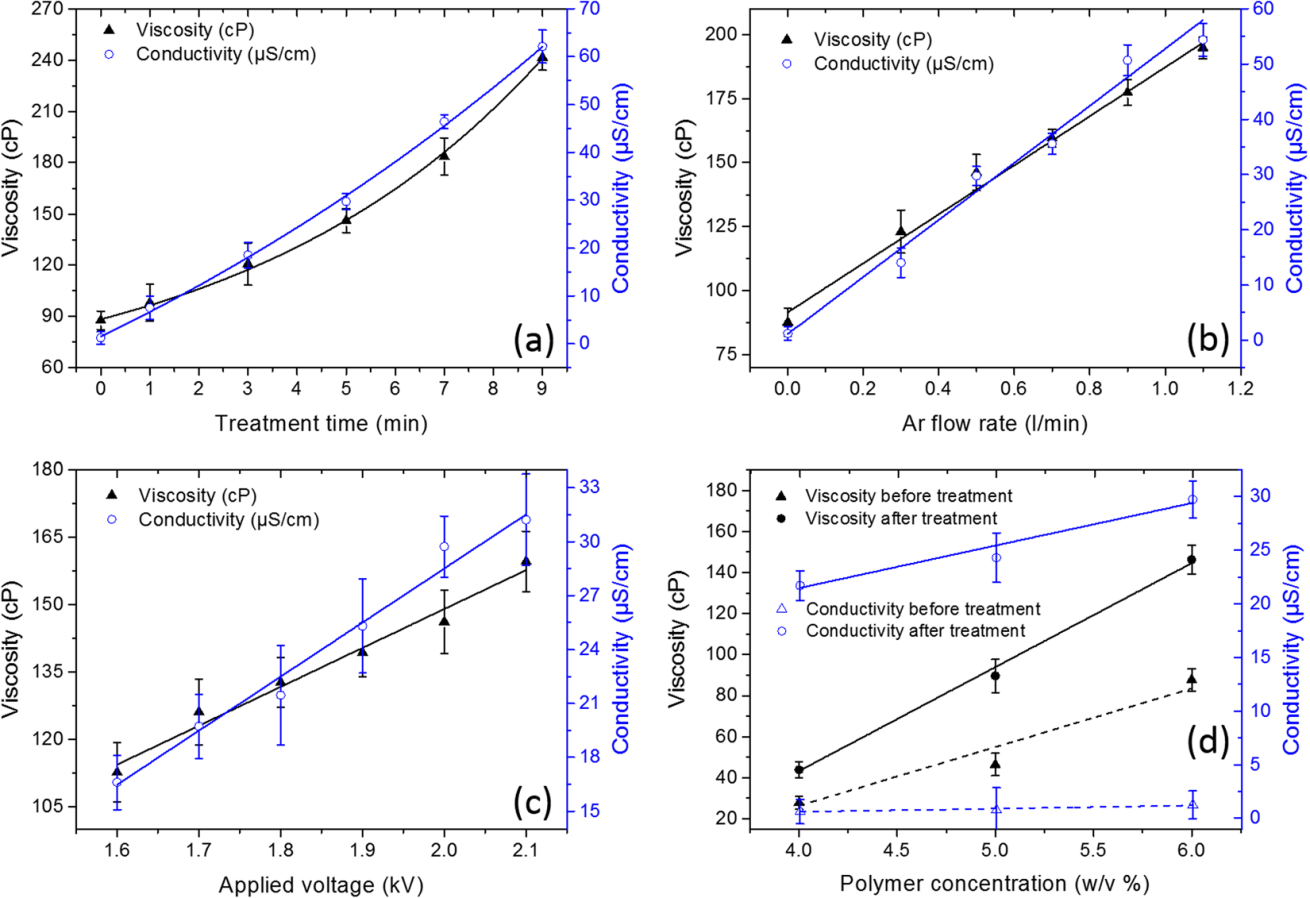
Viscosity and conductivity variations as a function of (**a**) treatment time (2 kV, 0.5 ${\rm{l}}\,{{\rm{\min }}}^{-1}$, 6% w/v), (**b**) argon flow rate (5 min, 2 kV, 6% w/v), (**c**) applied voltage (5 min, 0.5 ${{\rm{lmin}}}^{-1}$, 6% w/v), and (**d**) polymer concentration (5 min, 0.5 ${\rm{l}}\,{{\rm{\min }}}^{-1}$, 2 kV).

The viscosity and conductivity variations as a function of plasma treatment time, argon flow rate, applied voltage, and PLA concentration are plotted in Fig. 5. In each case, only a single plasma operational parameter was varied, while the other plasma parameters were fixed as mentioned in the caption of Fig. 5.

### Bubble Dynamics Inside the Plasma-Treated PLA Solutions

To be able to explain the effect of each individual plasma parameter on the viscosity and conductivity values (Fig. 5), information on the bubble formation in the PLA solutions will be revealed in this section as these bubbles mainly determine the occurring plasma-solution interactions. It has already been investigated by some research groups^24–26^ that charge, electron, and energy transfer reactions are possible at the plasma-liquid interface. Hence, more bubbles and consequently more gas-solution interfaces, cause an increase in collisional frequency of reactive plasma species with solvent and polymer molecules at the gas-solution interfaces, which will in turn lead to a better efficiency of the plasma treatment. Therefore, ICCD imaging will be carried out in the next section to study the effect of the plasma operational parameters on the bubble dynamics in the PLA solutions during plasma treatment.

In a first step, the bubble behavior inside the PLA solutions is examined as a function of plasma treatment time and the obtained results are presented in Fig. S1. The time points in these figures were chosen to be less than one minute as one minute after starting the plasma treatment a lot of bubbles were formed inside the system making it very difficult to observe differences between the various operational conditions. The effect of argon flow rate and applied voltage on the plasma bubble dynamics are presented in Figs S2 and S3, respectively. Moreover, bubble formation in different solutions used in this work was also examined and obtained ICCD images are presented in Fig. S4.

### Surface Tension of the Plasma-Treated PLA Solutions

Besides viscosity and conductivity, also the surface tension of a polymer solution is known to significantly influence the electrospinning process^13^. The surface tension values of the PLA solutions were therefore also measured before and after plasma treatment making use of different plasma operational parameters.

### Effect of Plasma Treatment on Nanofiber Morphology

**Figure 6:**
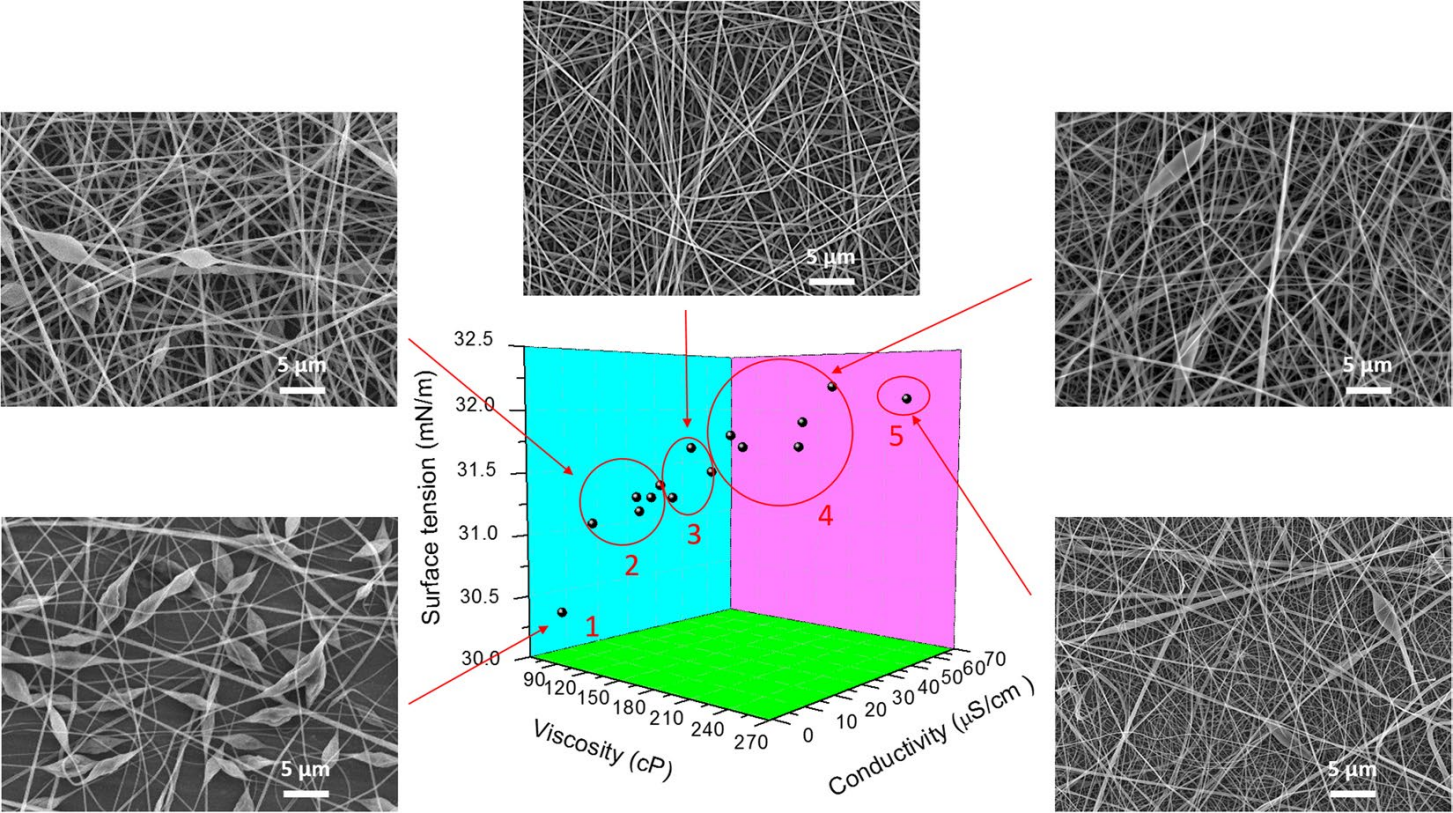
3D image of the physical properties of selected pristine and plasma-treated PLA solutions and SEM images of PLA nanofibers produced from these solutions (starting PLA concentration: 6% w/v).

As the three physical solution parameters (viscosity, surface tension, and conductivity) are considerably changed by the performed plasma treatments, it is anticipated that these plasma treatments will also have a significant effect on the morphology of the electrospun nanofibers produced from these solutions. To examine this, PLA solutions with varying values of viscosity, conductivity, and surface tensions were prepared in this work by performing plasma treatments with different operational parameters. A three-dimensional image indicating the physical properties of the PLA solutions is shown in Fig. 6 as well as representative SEM images of the selected PLA nanofibers produced from these solutions by electrospinning.

## Discussion

Figure 1a shows that the plasma dynamics are periodic. The discharge voltage has a sinusoidal waveform, whereas the current waveform has one strong peak every negative half cycle of the applied voltage. These strong current peaks are due to the rapid increase in gas conductivity at plasma ignition followed by an accumulation of surface charge acting to reduce the gas voltage and extinguish the discharge^27^. Also, the positive half cycles have a current peak of lower amplitude of about 10 mA compared to the negative half cycle. The Lissajous curve presented in Fig. 1b is representative for all experimental conditions used in this work and clearly shows that the discharge voltage and charge waveforms are symmetrical for both polarities of the applied voltage. From literature^28,29^, it is well known that the area of the Q-V curve corresponds to the energy input per cycle meaning that the discharge power can be calculated by multiplying this energy input with the driving frequency of the discharge (equation 2), which is equal to 50 kHz.

Figure 2 clearly shows that the mean power increases with applied voltage which is rather straightforward. Figure 2 also reveals that the mean power also increases with increasing flow rate, which is due to the fact that with increasing gas flow rate, the discharge voltage and discharge current increase consequently resulting in an increase in mean power as well^30^. The Lissajous method, based on integration of the current with the use of a capacitor, results in more accurate results compared to the V-I method where instantaneous power is measured^31^. The mean power values obtained from the Q-V method are lower than those obtained via the V-I method, however, the values only have a maximal difference of ±5.88%.

Table 1 and Fig. 3 show that the most predominant emission line, located at 308.49 nm, can be attributed to the rotational band of OH, while the large emission lines in the wavelength range 690–853 nm can be attributed to the atomic argon 4 s $\leftarrow \,$4p transitions. Additionally, small emission lines can be observed in the wavelength range 280–400 nm which can be assigned to nitrogen-containing species: excited nitrogen molecules (N_2_ second positive system, SPS) at 330–380 nm, ionized nitrogen molecules (${{\rm{N}}}_{2}^{+}$ first negative system, FNS) centered at 388 nm, atomic oxygen at 777.53 nm, and NO radicals at 282.87 nm. The nitrogen SPS peaks result from the lowest vibrational transition of the ${{\rm{B}}}^{3}{{\rm{\Pi }}}_{{\rm{g}}}\leftarrow {{\rm{C}}}^{3}{{\rm{\Pi }}}_{{\rm{u}}}$ system of ${{\rm{N}}}_{2}$ molecules^32^.

The identified nitrogen-containing components are most likely due to diffusion of environmental air into the plasma jet afterglow resulting into the presence of small amounts of ${{\rm{N}}}_{2}$ impurities and have been also detected by other research groups^33–35^. The OH emission peak is probably caused by fragmentation of ${{\rm{H}}}_{2}{\rm{O}}$ molecules which diffuse from ambient air to the plasma jet afterglow^32^, while the atomic oxygen emission peak can result from H_2_O fragmentation or O_2_ dissociation.

Apart from the detected chemical species and bands in the optical emission spectrum of the plasma jet operating in ambient air (Table 1), some extra transition lines for mostly hydrocarbon fragments appeared in the optical emission spectrum of the plasma-solution system (Fig. 4): a CN emission band (${{\rm{X}}}^{2}{{\rm{\Sigma }}}^{+}\leftarrow {{\rm{B}}}^{2}{{\rm{\Sigma }}}^{+}$) located at 416.13^36^, CH emission bands located at 386.89 nm (${{\rm{X}}}^{2}{\rm{\Pi }}\leftarrow {{\rm{B}}}^{2}{{\rm{\Sigma }}}^{-}$), and 431.19 nm (${{\rm{X}}}^{2}{\rm{\Pi }}\leftarrow {{\rm{A}}}^{2}{\rm{\Delta }}$)^32,37–39^, an NH band (${{\rm{X}}}^{3}{{\rm{\Sigma }}}^{-}\leftarrow {{\rm{A}}}^{3}{\rm{\Pi }}$) located at 336.52 nm^40^, Ar ($1{{\rm{s}}}_{4}\leftarrow 3{{\rm{p}}}_{5}$) located at 419.72 nm^41^, and the Swan system of ${{\rm{C}}}_{2}$ lines (${{\rm{X}}}^{3}{{\rm{\Pi }}}_{{\rm{u}}}\leftarrow {{\rm{A}}}^{3}{{\rm{\Pi }}}_{{\rm{g}}}$) located at 473.5, 516.29, 558.11, and 563.26 nm^32,42,43^. The presence of these additional peaks suggest that the PLA solution components are directly dissociated by the argon plasma jet^32,37,38,42^.

One of the other noticeable differences between the two optical emission spectra (Figs 3 and 4) is the decreased intensity of the OH peak. This decrease can be attributed to the high consumption of energy for CH and ${{\rm{C}}}_{2}$ bond cleavage, which consequently leads to having less energy available for OH radical production.

As can be seen in Fig. 5, viscosity and conductivity of the PLA solutions considerably increased with plasma treatment time (Fig. 5a), argon flow rate (Fig. 5b), applied voltage (Fig. 5c), and polymer concentration (Fig. 5d). The plots against gas flow, voltage, and polymer concentration show a linear relationship between the parameters viscosity and conductivity on the one hand and the variable plasma operational parameter on the other hand. However, for the plasma treatment time, exponential trends are obtained for both viscosity and conductivity.

The viscosity and conductivity measurements were also performed to study the plasma-induced changes in the solvents without the presence of PLA. Unfortunately, for pristine and plasma-modified CHL, DMF, and their binary mixture, the measured torque values were below the range of the viscometer device and thus viscosities were not measurable. The conductivity of the pristine CHL was very low and therefore not detectable with the used conductivity meter. Even after plasma treatment (5 min, 0.5 ${\rm{l}}\,{{\rm{\min }}}^{-1}$, 2 kV), the conductivity of the CHL solution was still undetectable. However, the conductivity of DMF alone was found to improve after plasma treatment (5 min, 0.5 ${\rm{l}}\,{{\rm{\min }}}^{-1}$, 2 kV) and strongly increased from 0.77 ± 0.1 ${\rm{\mu }}\,$S/cm for pristine DMF to 14.71 ± 0.2 ${\rm{\mu }}\,$S/cm for plasma-modified DMF. The conductivity of the binary-solvent system of CHL/DMF (8:2 v/v) also increased after plasma treatment from 0.53 ± 0.12 ${\rm{\mu }}\,$S/cm to 8.95 ± 0.2 ${\rm{\mu }}\,$S/cm. Based on these results, it is anticipated that the strong increases in solution conductivity observed after the performed plasma treatments are most likely resulting from interactions of active plasma species with DMF molecules. A possible hypothesis for the observed increased conductivity values after plasma treatments may be that plasma treatment induces some degradation of the solvent molecules^44,45^ and/or PLA polymer chains (as also suggested by the observed OES results) leading to the creation of chemical species with a high conductivity such as for example nitric acid (${{\rm{HNO}}}_{3}$), hydrogen chloride (HCl) or peroxynitrite (${{\rm{ONOO}}}^{-}$).

Besides an increase in solution conductivity, also the PLA solution viscosity was found to considerably increase after plasma modification as shown in Fig. 5. Within this context, it is important to highlight that the performed plasma experiments result in solvent evaporation, which can in turn significantly increase the PLA concentration in the solvent mixture. This increasing PLA concentration will mainly affect the solution viscosity and not the solution conductivity as can be seen in Fig. 5d. As shown in Table 2, the PLA solution volume strongly decreases with increasing plasma treatment time and as CHL is much more volatile than DMF it may be assumed that merely CHL has evaporated during the plasma treatment. To examine if this evaporation is mainly due to the argon gas flowing through the solution or due to the plasma treatment itself, the solution volume change was also investigated for polymer solutions which are only exposed to the argon flow without switching on the plasma for identical times as the plasma exposure times and the results are presented in Table 2.

**Table 2:** The final solution volume after plasma treatment (left) and after Ar-bubbling (right) with various exposure times (treatment condition: 0.5 ${\rm{l}}\,{{\rm{\min }}}^{-1}$, 2 kV, 6% w/v).

Plasma treatment		Ar-bubbling	
Time (min)	Final solution volume (ml)	Time (min)	Final solution volume (ml)
0	10	0	10
1	9.1	1	9.3
3	8.7	3	9
5	8.4	5	8.8
7	8.1	7	8.6
9	7.9	9	8.4

As shown in this table, the argon gas flow is causing solvent evaporation, however, this evaporation becomes more enhanced when the plasma is turned on. One may think that this enhanced evaporation effect is due to heating of the polymer solution by the plasma, however, this is not the case as the temperature of the PLA solutions is not raised by the plasma treatments. The enhanced solvent evaporation is however the result of more intense bubble formation in the plasma-exposed solutions leading to larger liquid-gas interfaces and thus a more pronounced evaporation, which has been confirmed via ICCD imaging (Fig. S5).

To examine whether the observed viscosity increase is the result of the plasma treatment itself and not of solvent evaporation, an additional experiment has been conducted in this work. In this experiment, 10 ml of a 6% w/v PLA solution was only exposed to the flowing argon gas (0.5 ${\rm{l}}\,{{\rm{\min }}}^{-1}$) without switching on the plasma for durations varying between 2.5 and 15.5 min until the same final solution volumes as after plasma treatment were reached. For these Ar-bubbled PLA solutions, the viscosity was also determined and the results are shown in Table 3, together with the viscosity results obtained on the plasma-treated PLA solutions. Based on the observed volume changes, the final PLA concentration in each solution has been calculated and the results are also shown in Table 3. As such, the effect of polymer concentration on solution viscosity can be eliminated and the plasma treatment effect can be observed. Table 3 shows that the plasma treatment itself is also increasing the solution viscosity and this mainly at the highest plasma exposure time.

**Table 3:** The final PLA concentration and obtained viscosity of the solutions after plasma treatment and Ar-bubbling with various exposure times (treatment condition: 0.5 lmin^−1^, 2 kV, 6% w/v).

PLA concentration (w/v %)	T_plasma_ (min)	T_bubbling_ (min)	Plasma treatment	Ar-bubbling
			Viscosity (cP)	Viscosity (cP)
6	0	0	87.72 ± 5.46	87.72 ± 5.46
6.6	1	2.5	98.04 ± 10.86	90.00 ± 6.48
6.9	3	5	120.80 ± 12.33	103.88 ± 8.34
7.1	5	9	146.16 ± 7.01	129.52 ± 4.61
7.4	7	12	183.58 ± 11.01	166.23 ± 7.44
7.6	9	15.5	241.52 ± 7.31	204.17 ± 8.52

The above mentioned hypothesis about the generation of chemical species with a high conductivity such as ${{\rm{HNO}}}_{3}$, HCl or peroxynitrite (${{\rm{ONOO}}}^{-}$) may also explain the increased viscosity after plasma treatment because these species are highly polar and can as such increase the solubility of PLA in the solvent mixture. As a result, the distribution of PLA polymer chains in the solution becomes more uniform, which can in turn explain the observed enhanced viscosity after plasma modification.

ICCD images (Fig. S1) show that with increasing plasma treatment time, more bubble formation occurs in the plasma-liquid system, which can positively affect the efficiency of the plasma treatment. Besides the larger frequency of collisions between the plasma species and the solution, also the total contact time between the active plasma species and the polymer/solvent molecules increases with increasing treatment time, which in turn also enhances the plasma treatment effect. Both aspects thus contribute to more pronounced interactions between active plasma species and the PLA solution, thereby resulting in increasing viscosity and conductivity values as a function of plasma treatment time.

The effect of argon flow rate on the plasma bubble dynamics is presented in Fig. S2. One can clearly see that the bubble number density considerably increases with increasing gas flow rate resulting in more pronounced plasma-liquid interactions and thus a more efficient plasma treatment effect. At the same time, it is also well known that the electron number density in the plasma jet increases when the gas flow rate raises^46^. Consequently, more active plasma species will be generated in the plasma, which can in turn result in more intense interactions between the plasma and the solution molecules. Because of the two previously mentioned reasons, increased viscosity and conductivity values can indeed be expected at increased gas flow rates.

ICCD images visualizing the plasma bubble dynamics for two different applied voltages are shown in Fig. S3. These images reveal that with increasing applied voltage more bubbles appear which also have the tendency to coalescence. As a result, a larger amount of plasma-solution collisions will occur at higher applied voltages, which will in turn enhance the plasma treatment effect in terms of solution viscosity and conductivity. Additionally, an increase in applied discharge voltage is known to result in a higher amount of active plasma species^47^ and thus more intense interactions between plasma species and solution molecules. Both bubble dynamics and plasma species concentration thus contribute to an enhanced plasma treatment efficiency, thereby leading to increased viscosity and conductivity values at higher applied voltages.

In a next set of experiments, bubble formation was examined in different solutions: (1) pure CHL, (2) pure DMF, (3) the solvent mixture CHL/DMF, (4) a 4% w/v PLA solution in the solvent mixture, and (5) a 6% w/v PLA solution in the solvent mixture and the obtained ICCD images are shown in Fig. S4.

Since pure CHL is a non-polar solution characterized by a low dielectric constant (see Table 4), no bubble formation occurred during the plasma treatment as can be observed in Fig. S4a. Instead, gas flow-induced foams were formed on top of the solution which also disappeared immediately after plasma treatment. The non-polar nature of CHL combined with its low dielectric constant thus results in a rather poor interaction between the plasma species and the CHL molecules, which may also explain the fact that no detectable increase in solution conductivity was observed for pure CHL. On the other hand, a few small bubbles are formed in a pure DMF solution as shown in Fig. S4b which can be explained by the fact that DMF is a polar solution with a much higher dielectric constant compared to CHL (see Table S1). As such, it can be expected that more pronounced plasma-induced changes can occur in the pure DMF solution, which is in agreement with the observed increase in DMF conductivity after plasma treatment. Due to the presence of a small amount of DMF in the solvent mixture solution (CHL/DMF 8:2 v/v), the solvent mixture is only slightly polar and not as conductive as pure DMF. As a result, bubble formation in the solvent mixture is less than in pure DMF, as can be visualized in Fig. S4c, however, it is still enough to considerably enhance the conductivity of the mixture solution after plasma treatment.

**Table 4:** The surface tension of PLA solutions at various treatment times (2 kV, 0.5 l min^−1^, 6% w/v), argon flow rates (5 min, 2 kV, 6% w/v), and applied voltages (5 min, 0.5 lmin^−1^, 6% w/v).

Treatment time (min)	Surface tension (mN/m)	Argon flow (lmin^−1^)	Surface tension (mN/m)	Voltage (kV)	Surface tension (mN/m)
0	30.4 ± 0.08	0	30.4 ± 0.08	1.6	31.3 ± 0.07
1	31.1 ± 0.08	0.3	31.2 ± 0.08	1.7	31.4 ± 0.12
3	31.3 ± 0.05	0.5	31.5 ± 0.09	1.8	31.3 ± 0.10
5	31.5 ± 0.09	0.7	31.7 ± 0.09	1.9	31.7 ± 0.10
7	31.7 ± 0.07	0.9	31.9 ± 0.16	2.0	31.5 ± 0.09
9	32.1 ± 0.10	1.1	32.2 ± 0.17	2.1	31.8 ± 0.14

ICCD imaging also reveals that when PLA is added to the solvent mixture, bubble formation becomes much more pronounced. In addition, when the PLA concentration is increased from 4 to 6% w/v, more bubble nucleation and bubble formation occurs inside the solution. Because of these different bubble dynamics, increased viscosity and conductivity values can be expected at increased polymer concentrations, as was indeed observed in Fig. 5d.

Table 4 clearly shows that the surface tension of the PLA solutions increases with increasing treatment time, increasing argon flow rate and increasing applied voltage, which are similar trends as the ones observed for viscosity and conductivity.

To examine whether these observed increases in surface tension are induced by the plasma or caused by solvent evaporation, a similar evaporation experiment as described in the section on the viscosity results was also performed. For the Ar-bubbled PLA solutions, the surface tension was determined and the results are shown in Table 5 together with the surface tension results obtained on the plasma-treated solutions for an easy comparison. As such, the effect of polymer concentration on the surface tension values can be eliminated and the plasma treatment effect can be observed.

**Table 5:** The final PLA concentration and obtained surface tension of the solutions after plasma treatment and Ar-bubbling with various exposure times (treatment condition: 0.5 lmin^−1^, 2 kV, 6% w/v).

PLA concentration (w/v %)	T_plasma_ (min)	T_bubbling_ (min)	Plasma treatment	Ar-bubbling
			Surface tension (mN/m)	Surface tension (mN/m)
6	0	0	30.4 ± 0.08	30.4 ± 0.08
6.6	1	2.5	31.1 ± 0.08	30.7 ± 0.09
6.9	3	5	31.3 ± 0.05	30.7 ± 0.10
7.1	5	9	31.5 ± 0.09	29.8 ± 0.12
7.4	7	12	31.7 ± 0.07	30.5 ± 0.09
7.6	9	15.5	32.1 ± 0.10	30.9 ± 0.11

The results shown in Table 5 reveal that the observed increase in the final surface tension of the PLA solutions is not caused by evaporation but is mostly an effect of the plasma treatment itself. As such, it can indeed be concluded that the surface tension of the PLA solutions increases with increasing treatment time, argon flow rate, and applied voltage.

ICCD images (Figs S1, S2, and S3) showed that the bubble number density increases with increasing treatment time, gas flow rate, and applied voltage. Moreover, it has also been reported^24^ that increasing flow rate and increasing applied voltage lead to an increase in charge and energy transfers from a plasma jet to a liquid. Because of the enhanced bubble dynamics resulting into more plasma-liquid interactions as well as the enhanced energy transfer between the two media, surface tension values will thus increase with increasing plasma operational parameters. In addition, the higher bubble densities will also result in a higher charge transfer from the plasma to the polymer solutions which will in turn contribute to a change in surface tension at the gas-solution interfaces^48^. At higher charge densities, relatively more depletion of charges within the surface of the solutions occurs and the bulk charge density increases, supplementing the attractive forces on the surface molecules, and consequently adding to an increase in surface tension^49^.

The surface tension values of pure CHL, DMF and their binary mixture have also been examined before and after plasma modification and the results are shown in Table 6 together with surface tension values for PLA solutions with various PLA concentrations. This table clearly shows that in contrast to the PLA solutions, the surface tension of the solvents decreased after plasma modification. This reduction may be due to the surface-active nature of the plasma-induced uncharged acids (such as HCl, HNO_3_, NH_3_) present in the solvents^49^. Table 6 also reveals that when PLA is added to the binary-solvent mixture, the surface tension increases and the higher the added PLA concentration, the higher the surface tension value becomes. This can be explained as follows: according to the Gibbs adsorption rule^48^, when PLA is added to the binary-solvent system, more charges repel from the gas-liquid surface and the bulk charge density increases, consequently resulting into an increased surface tension.

**Table 6:** The surface tension of the solvents and PLA solutions with different polymer concentrations (5 min, 0.5 lmin^−1^, 2 kV).

Solution	Surface tension (mN/m)	
	Pristine	Plasma-modified
CHL	27.2 ± 0.12	26.4 ± 0.11
DMF	36.7 ± 0.04	36.5 ± 0.07
CHL/DMF (8:2 v/v)	28.8 ± 0.12	28.3 ± 0.12
PLA/mixture (4% w/v)	29.1 ± 0.03	29.7 ± 0.06
PLA/mixture (5% w/v)	29.8 ± 0.03	30.4 ± 0.03
PLA/mixture (6% w/v)	30.4 ± 0.08	31.5 ± 0.09

As can be observed from the SEM images in Fig. 6, different fiber morphologies were captured for different value ranges of the three examined physical parameters. For solution 1, which is a pristine PLA solution (6% w/v), polymeric beads were obtained along the PLA nanofibers. For this solution, electrospraying overcomes the electrospinning process because of the low viscosity (due to the low PLA concentration) and the high surface tension of the pristine PLA solution^50^, which is known to result in the formation of beads. After performing plasma treatments resulting into slightly increased viscosity, conductivity, and surface tension values, a better fiber formation and less beads along the fibers were observed (region 2). Nevertheless, the beads did not completely disappear in this region. After electrospinning PLA solutions with slightly higher viscosity, conductivity, and surface tension values compared to the solutions of region 2, the morphology of the deposited nanofibers changed to very homogenous and smooth fibrous networks (region 3). In this region, PLA nanofibers with small fiber diameters (314 ± 58 nm), a uniform fiber distribution and without the presence of beads were obtained. As a result of the higher viscosity values, there is a higher amount of polymer chains entanglement in the solution and the charges on the electrospinning jet will be able to fully stretch the solution with the solvent molecules distributed among the polymer chains. In addition, due the higher solution conductivity, more charges can be carried by the electrospinning jet and a better stretching of the solution will also occur. Because of the enhanced stretching effect, nicely elongated fibers without the presence of beads can thus be obtained^13,23^.

However, with a further increase in conductivity, viscosity, and surface tension values (region 4), the beads again appeared while the diameter of the PLA nanofibers also increased most likely due to the high viscosity values. Moreover, for the extreme physical PLA solution parameters obtained in this work (region 5), beaded, deformed, and non-uniform fibers were observed. Discontinuities of the fibers were also found in this condition as a result of clogging during the electrospinning process due to the high solution viscosity, which leads to a decreased productivity of the process and also negatively affects the nanofibers quality^51^. It can thus be concluded that plasma-treated PLA solutions with physical parameters located in region 3 are optimal to generate homogeneous, bead-free PLA nanofibers.

**Figure 7:**
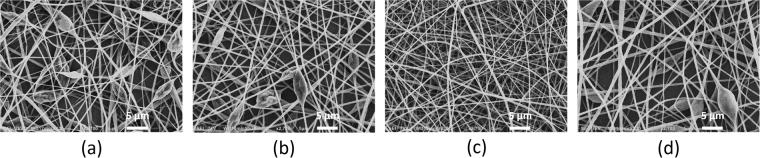
SEM images of PLA nanofibers produced from (**a**) pristine PLA solution in CHL/DMF (6% w/v), (**b**) an Ar-bubbled 6% w/v PLA solution (5 min bubbling), (**c**) a plasma-modified 6% w/v PLA solution (5 min, 2 kV, 0.5 lmin^−1^), and (**d**) an Ar-bubbled 6% w/v PLA solution (9 min bubbling).

To further show that the plasma treatment effect on the electrospinnability of PLA solutions was not only due to the solvent evaporation it is causing, an additional experiment was also performed in this work. In this experiment, four different PLA solutions have been electrospun and the morphology of the resultant PLA nanofibers was observed: (1) a pristine 6% w/v PLA solution, (2) a 6% w/v PLA solution which was exposed to the flowing argon gas (0.5 lmin^−1^) in plasma-off mode for a duration of 5 min, (3) a 6% w/v PLA solution which was exposed to the plasma for 5 min (0.5 lmin^−1^ gas flow rate and 2 kV) having a final polymer concentration of 7.1% w/v and (4) a 6% w/v PLA solution which was exposed to the flowing argon gas (0.5 lmin^−1^) in plasma-off mode for a duration of 9 min to reach the same final polymer concentration as after the 5 min plasma treatment (7.1% w/v). The SEM images of the PLA nanofibers obtained from the four above mentioned PLA solutions are shown in Fig. 7.

This additional experiment clearly reveals that the obtained changes in the morphology of PLA nanofibers fabricated from plasma-modified PLA solutions cannot be attributed to Ar-bubbling nor to the change in polymer concentration due to solvent evaporation. In the Ar-bubbling experiment of 5 min, argon gas and PLA solution mixing occurs, however, the neutral argon gas components are not able to effectively interact with the PLA solution and only a small enhancement in viscosity of the solution was obtained after this experiment. This small change in viscosity is not sufficient to generate bead-free fibers as can be seen in Fig. 7b. Additionally, if a PLA solution with the same final polymer concentration as the plasma-treated solution is electrospun, the obtained PLA nanofibers still contain beads despite the higher polymer concentration and consequently the increased viscosity (see Fig. 7d). Only after plasma treatment and due to a considerable enhancement in both solution viscosity and conductivity, bead-free and uniform PLA nanofibers could be obtained as can be seen in Fig. 7c. As such, plasma treatments are thus very efficient in improving the electrospinnability of PLA as they have the capacity to affect different physical solution parameters at the same time.

## Methods

### Sample Polymer Solution Preparation

PLA ((C_3_H_4_O_2_)_n_, M_w_ ~ 230.000 g/mol) was purchased from Goodfellow and used as target polymer in this work. Chloroform (CHL; Sigma Aldrich, 99.5%) and N,N-Dimethylformamide (DMF; Sigma Aldrich, 99.8%) were used as solvents for preparation of electrospinning solutions. PLA granules were added to a binary-solvent system of CHL/DMF (8:2 v/v) to create polymer solutions with concentrations ranging from 4 to 6% w/v. The mixtures were magnetically stirred at room temperature until complete dissolution of the polymer granules to prepare uniform and transparent PLA solutions.

### Experimental Set-ups

#### Plasma Set-up

**Figure 8:**
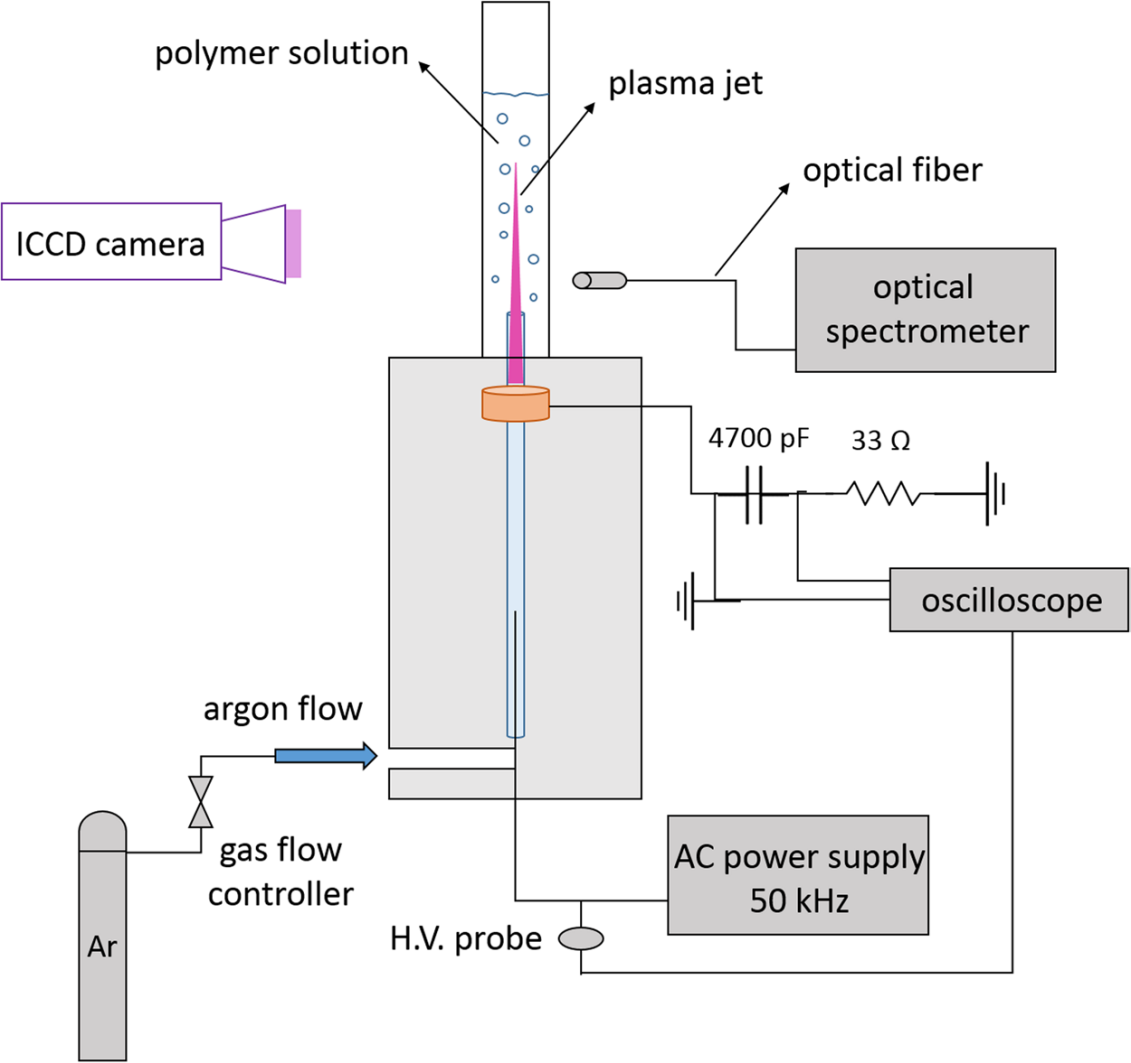
Schematic diagram of the submerged atmospheric-pressure argon plasma jet for polymer solution treatment.

An atmospheric-pressure submerged plasma jet, depicted in Fig. 8, was used to modify PLA solutions before electrospinning. The argon plasma jet was composed of a cylindrical quartz tube (inside and outside diameters of 1.5 and 3 mm respectively and 130 mm long) and an aluminum rod as high-voltage electrode, which was embedded inside the quartz tube. The quartz tube was covered by a Teflon shell (which was a hollow cylinder with inside and outside diameters of 13 and 62 mm, respectively) and was aligned along the central axis of this Teflon cylinder. The high-voltage electrode was connected to a 50 kHz sinusoidal customer-made power supply with maximum output voltage and power of 25 kV (peak to peak) and 500 W, respectively. A copper ring (∅=1  mm, thickness = 10.5 mm) was fixed 18 mm away from the tip of the high-voltage pin electrode and serves as a ground electrode. The distance between the bottom of the copper ring and the end of the capillary is 40 mm. Argon (Air Liquid, purity >99.999%) was used as carrier gas in this work and the gas flow rate through the capillary was controlled by a mass flow controller (Model: F-201CV, Bronkhorst, Netherlands). The sample holder for polymer solutions was a quartz tube with an inner and outer diameter of 17.5 and 22.5 mm, respectively which was placed around the high end of the plasma jet quartz tube as schematically shown in Fig. 1. Prior to plasma modification, the quartz sample holder is filled with 10 ml of the PLA solution and this volume is kept constant in all experiments. As such, the liquid height in the quartz sample holder was each time approximately 40 mm. Several plasma operational parameters were varied in this study including plasma exposure time (1–9 min), amplitude of the applied voltage (1.6–2.1 kV), argon flow rate (0.3–1.1 lmin^−1^) while also the polymer concentration of the PLA solutions will be changed between 4 and 6% w/v.

#### Electrospinning Set-up

**Figure 9:**
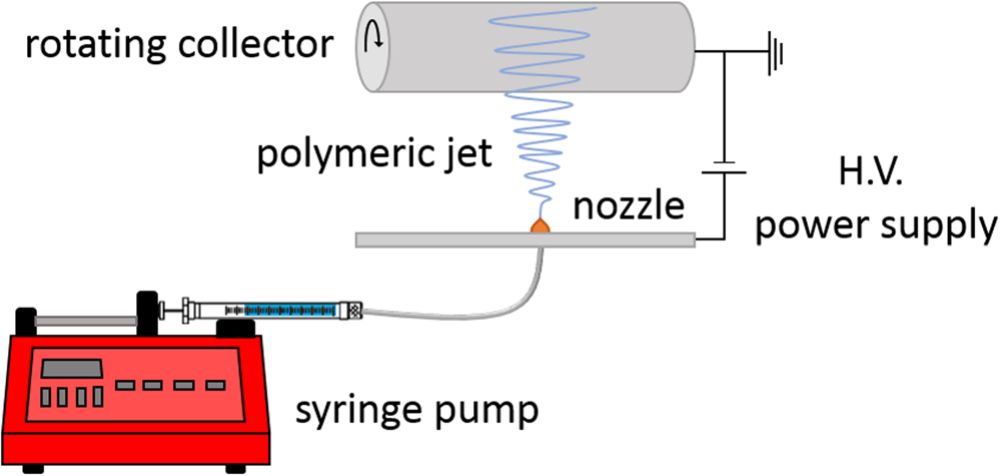
Schematic representation of the electrospinning equipment used to electrospin untreated and plasma-modified PLA solutions.

The electrospinning process was performed on a standard electrospinning apparatus with a bottom-up configuration (Nanospinner 24, Inovenso, Turkey) as schematically shown in Fig. 9. Untreated and plasma-modified PLA solutions were collected in a 10 ml plastic syringe and for the plasma-modified samples, collection is performed immediately after plasma modification. The filled syringe was subsequently placed in a syringe pump (NE-300 Just Infusion™ syringe pump) which controls the flow rate of the polymer solution through a polyethylene tube (inner diameter of 2 mm) ending in an aluminum feeding pipe containing a single brass nozzle with an inner diameter of 0.8 mm. During the electrospinning process, the polymer flow rate is maintained at 1 ml/h. The brass nozzle is vertically placed below a stainless steel drum collector rotating at 100 rpm at a distance of 17.5 cm. During the electrospinning process, a DC high voltage of 23 kV is applied to the nozzle, while the cylindrical collector is grounded. In this work, nanofibers are directly collected on an aluminum foil covering the rotating drum and the electrospinning process was carried out at ambient temperature with a relative humidity varying between 50 and 60%.

### Electrical and Optical Characteristics of the Plasma Jet

The electrical parameters such as discharge voltage, discharge current, and mean consumed power were studied for the argon plasma jet used in this work at different flow rates and applied voltages. The power consumed by the discharge was calculated using two methods: (1) using the traditional method which multiplies the discharge voltage with the current, and (2) using Lissajous curves. For the first method, the voltage applied to the wire electrode was measured using a high voltage probe (P6015A, Tektronix), whereas the discharge current was obtained by measuring the voltage over a non-inductive resistor of 33 ${\rm{\Omega }}$ placed in series with the plasma jet (see Fig. 8). The resultant voltage-current waveforms were subsequently recorded making use of a Tektronix TDS 1002 digital oscilloscope. For the second method, the voltage applied to the wire electrode was measured using the same high voltage probe as mentioned before whereas the charge present on the electrodes was also obtained by measuring the voltage over a 4700 pF capacitor placed in series with the plasma jet (see Fig. 8) The resultant charge-voltage waveforms (so-called Lissajous figures) are again recorded making use of a Tektronix TDS 1002 digital oscilloscope.

Besides the above mentioned electrical characterization, the plasma jet was also optically investigated. Optical emission of the argon plasma jet was monitored by an optical spectrometer (Ocean Optics, ADC1000-USB) with low spectral resolution of 0.7 nm in the wavelength range from 200 to 900 nm to gather information on the (excited) radiative plasma species present in the discharge.

### ICCD Imaging of the Bubble Dynamics

Dispersed time- and space-resolved emission pattern images of bubble dynamics in the argon plasma-solution system were obtained using a fast gated ICCD camera (Model: C8484, Hamamatsu, Japan) with UV optics with high transparency above 270 nm. A light emitted diode (Model: M530L3, Thorlabs, Germany) was used for background illumination of the discharge and the bubbles inside the polymer solution. A single shoot mode with no synchronization in between the ICCD camera gating unit and the plasma source was applied due to unrepeatable behavior of the discharge and the exposure time was fixed at 10 ms for all imaging measurements.

### Physical Characterization of the PLA Solutions

To characterize the PLA polymer solution, several important physical solution parameters which are known to influence the electrospinning process are measured before and immediately after each conducted plasma treatment: (1) viscosity, (2) conductivity and (3) surface tension. All measurements were performed at least three times for each experimental condition; mean values and standard deviations were calculated and will be reported in this study. Solution viscosity was obtained using a DV2T EXTRA viscometer (Brookfield Engineering Laboratories, USA) operating at room temperature. Solution electrical conductivity measurements were carried out using a FiveEasy^TM^ conductivity meter (Mettler Toledo, Switzerland) equipped with an InLab720 conductivity probe operating in a conductivity range of 0.1 to 500 μS/cm. The surface tension of each solution was determined using a K20 Easy Dyne Tensiometer (Krüss GmbH, Germany) equipped with a Wilhelmy plate at room temperature. The system records the force required to break the plate away from the solution surface. The surface tension was then calculated from the following formula:

3 $${\rm{\gamma }}=\frac{{\rm{F}}}{{\rm{L}}\times \,\cos \,{\rm{\theta }}}$$

where ${\rm{\gamma }}$ is the surface tension (mN/m), F is the maximum force acting on the plate (mN), L is the wetted length (m) and θ is the wetting angle. Assuming that the plate is completely wetted, the contact angle θ is equal to 0°, which means that cos θ is equal to 1. Therefore, the measurement of the surface tension is only affected by the measured force and the wetted length.

### Characterization of Electrospun PLA Nanofibers

The morphology of the electrospun nanofibers is imaged making use of an InTouch Scope JSM-6010 SEM device (JEOL, Belgium). The SEM images are acquired with an accelerating voltage of 7 kV and a working distance of 11 mm, after sputter-coating the samples with a thin layer of gold with a JFC-1300 Auto Fine Coater (JEOL, Belgium).

## Conclusion

In this study, a novel directly submerged argon plasma jet has been applied to treat PLA pre-electrospinning solutions in an effort to enhance PLA electrospinnability. The performed plasma treatments were found to enable the production of smooth, uniform, bead-free PLA nanofibers which could not be developed making use of the untreated PLA solutions under study. The improved PLA electrospinnability can be mainly attributed to a plasma-induced increase in solution conductivity in combination with an increase in solution viscosity. The influence of various plasma parameters such as plasma treatment time, argon flow rate, applied voltage as well as the influence of PLA concentration were thoroughly investigated and were found to have a significant effect on the final PLA nanofiber morphology as well as on the polymer solution physical properties. Bubble formation during the plasma treatments was also found to play a key role as it strongly influences the gas-solution interfaces and as a result also the charge and energy transfer possibilities between the plasma and the solutions. Good correlations between the plasma bubble behavior and the obtained physical properties of the PLA solutions were revealed. In the near future, the possible plasma-induced chemical changes in the pre-electrospinning PLA solutions will be examined in detail^52^.

## Electronic supplementary material

Supplementary Information
