# Supplementary material for: Plasma Modification of Poly Lactic Acid Solutions to Generate High Quality Electrospun PLA Nanofibers
Source: Sci Rep. 2018 Feb 2;8:2241. doi: 10.1038/s41598-018-20714-5 (PMC5797159; doi:10.1038/s41598-018-20714-5)
Supplement: Supplementary file 1 — Supplementary Information [file 41598_2018_20714_MOESM1_ESM.pdf]

Supporting Information

Plasma Modification of Poly Lactic Acid Solutions to Generate High Quality Electrospun PLA Nanofibers

Fatemeh Rezaei[1], Anton Nikiforov[1], Rino Morent[1], Nathalie De Geyter[1]

[1] Research Unit Plasma Technology (RUPT), Department of Applied Physics, Faculty of Engineering and Architecture, Ghent University, St-Pietersnieuwstraat 41 B4, 9000 Ghent, Belgium

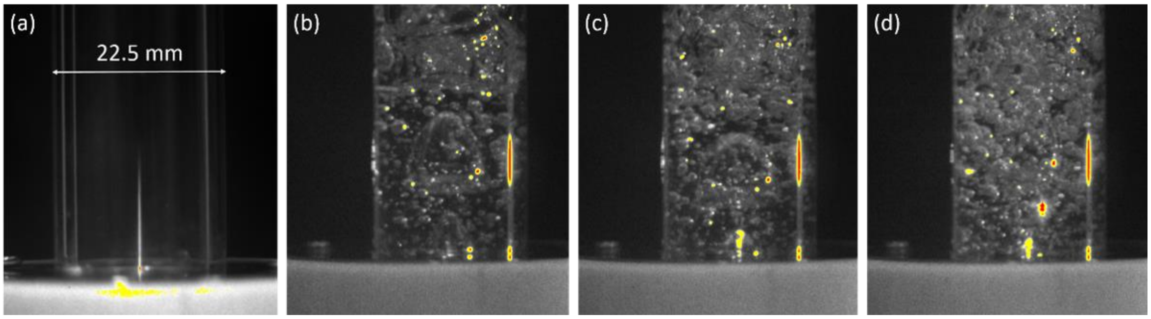

Fig. S1. (a) Pure argon plasma jet (1.8 kV, 0.5 $\mathrm{lmin^{-1}}$). Variation of the number density and size of bubbles at different treatment times; (b) 10 s, (c) 25 s, and (d) 55 s. (For all experiments: 6% w/v, 0.5 $\mathrm{lmin^{-1}}$, 1.8 kV)

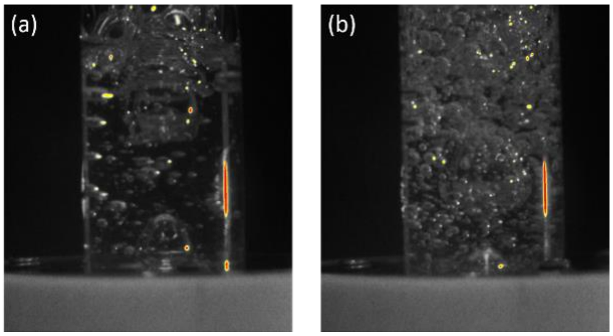

Fig. S2. Variation of the number density and size of bubbles at different flow rates; (a) 0.3 $\mathrm{lmin^{-1}}$, and (b) 0.5 $\mathrm{lmin^{-1}}$. (For all experiments: 6% w/v, 1.8 kV, 20 s)

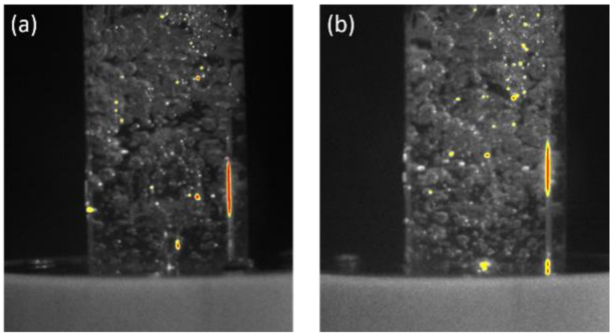

Fig. S3. Variation of the number density and size of bubbles at different applied voltages; (a) 1.8 kV, and (b) 2.1 kV. (For all experiments: 6% w/v, 0.5 $\mathrm{lmin^{-1}}$, 20 s)

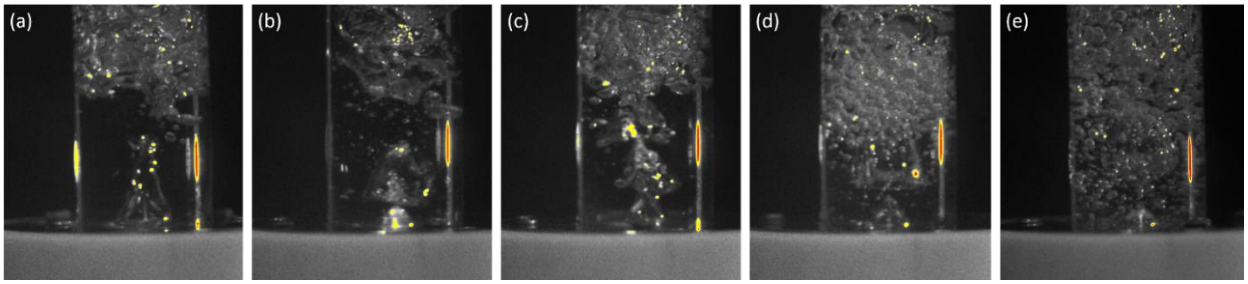

Fig. S4. Variation of the number density and size of bubbles in different solutions. (a) CHL, (b) DMF, (c) CHL/DMF (8:2 v/v), (d) 4% w/v PLA in the solvent mixture, and (e) 6% w/v PLA in the solvent mixture. (For all experiments: 1.8 kV, 0.5 $\mathrm{lmin^{-1}}$, 20 s)

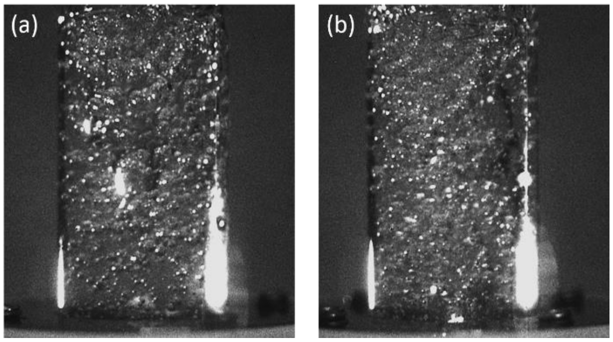

Fig. S5. Difference between the number density of bubbles in (a) Ar-bubbled PLA solution (6% w/v, 0.5 lmin^{-1}) and (b) plasma-treated PLA solution (6% w/v, 0.5 lmin^{-1}, 1.8 kV) at the same time scale.

Table S1. Dielectric constant ε of the solvents.

**Solvent**	**Chemical formula**	**Dielectric constant (F/m)**
CHL	$CHCl_3$	4.81
DMF	C_3H_7NO	36.7
